# Comparison of myocardial cooling effects between antegrade and retrograde cardioplegia: a retrospective study using thermography

**DOI:** 10.1186/s13019-025-03814-y

**Published:** 2025-12-28

**Authors:** Yoshiyuki Nishimura, Go Kuwahara, Hideichi Wada

**Affiliations:** 1 Department of Cardiovascular Surgery, Mie Heart Center, Mie, Japan; 2 Department of Cardiovascular Surgery, Fukuoka University, 7-45-1 Nanakuma, Jonan-ku, Fukuoka, 814-0180 Japan

**Keywords:** Myocardial protection, Cardioplegia, Thermography, Antegrade perfusion, Retrograde perfusion

## Abstract

**Background:**

Cardioplegia is delivered via antegrade, retrograde, or combined perfusion routes. Antegrade cardioplegia follows the physiological path but may be less effective in patients with severe coronary disease. Retrograde delivery allows continuous surgical manipulation but may result in uneven myocardial protection. This study compared myocardial cooling effects across delivery methods using thermographic imaging.

**Methods:**

Of 221 open-heart surgeries at our institution between May 2020 and November 2023, 22 cases with thermographic myocardial temperature data were retrospectively analyzed. All procedures were performed using blood cardioplegia (MPS®2, Quest. Medical, Inc.) Twenty-two patients initially received antegrade cardioplegia. For the second dose, they were divided into Group A (antegrade, n = 10) and Group R (retrograde, n = 12). Myocardial surface temperatures were assessed at multiple time points using thermography.

**Results:**

At the start of initial cardioplegia, myocardial surface temperatures were 31.6 ± 0.7°C in Group A and 31.8 ± 1.4°C in Group R, with infusion volumes of 2050 ± 396 mL and 2072 ± 251 mL, respectively; differences were not significant. Following the first dose (20°C cardioplegia), temperatures dropped to 22.7 ± 0.8°C and 23.8 ± 1.6°C, respectively, with no significant difference due to identical delivery. At the second dose, temperatures were 27.6 ± 1.2°C in Group A and 28.8 ± 1.7°C in Group R. After the second dose, Group A showed significantly lower temperatures (22.7 ± 0.6°C) compared to Group R (24.6 ± 1.9°C, p < 0.05). No intergroup differences were seen in postoperative ejection fraction, creatine kinase, catecholamine use, extubation time, hospital stay, or overall clinical outcomes. Cardiac function was preserved in both groups.

**Conclusion:**

While retrograde cardioplegia facilitates uninterrupted procedures, it may be less effective for myocardial cooling, raising concerns about protective adequacy. Thermography offers a practical, noninvasive means to assess myocardial temperature and detect insufficient cooling, enhancing myocardial protection strategies.

## Background

Hypothermic chemical cardioplegia combines chemical cardiac arrest, hypothermia, and adjunctive myocardial protection and has been shown to extend the safe duration of myocardial ischemia. Chemical cardiac arrest reduces myocardial oxygen consumption to about one-tenth of baseline, and further cooling lowers it to roughly one-half to one-third of the already reduced level [1, 2].

Traditionally, myocardial temperature has been measured by inserting a thermistor needle into the interventricular septum to assess the effectiveness of myocardial protection [3, 4]. However, this method is invasive, provides only localized temperature data, and is now less commonly used due to the widespread standardization of cardioplegia techniques. Nevertheless, accurately assessing myocardial temperature remains essential for evaluating the adequacy of myocardial protection. We have previously reported the utility of thermography, a noninvasive method for monitoring temperature during myocardial protection [5].

Cardioplegic solutions may be delivered via antegrade perfusion, retrograde perfusion, or a combination of both, with the choice of method varying by institution. Antegrade perfusion follows the physiological route, delivering the solution through a needle inserted into the aortic root or selectively into the left and right coronary ostia. However, in patients with severe coronary artery disease, myocardial cooling may be inadequate or uneven. In contrast, retrograde perfusion delivers the solution through a catheter inserted into the coronary sinus, allowing myocardial protection without interrupting surgical manipulation. Although this method facilitates continuous surgery, it may not achieve uniform cooling of the entire myocardium.

This study aimed to compare the effects of antegrade and retrograde cardioplegia on myocardial cooling using thermographic imaging.

## Methods

Total 221 patients who underwent open-heart surgery at our institution between May 2020 and November 2023, those whose intraoperative myocardial surface temperature was recorded using thermography were retrospectively included. All procedures were performed using blood cardioplegia (MPS^®^2, Quest. Medical, Inc.) based on a Buckberg-type formula, administered at a delivery temperature of 20 °C and were performed in an operating room maintained between 22 and 24 °C. Patients were managed under mild systemic hypothermia (core temperature 32–34 °C) throughout cardiopulmonary bypass. All patients received an initial dose of cardioplegia via antegrade coronary perfusion to induce cardiac arrest. At our institution, the second cardioplegia dose is routinely administered approximately 25 min after the first dose, or earlier if myocardial movement begins in the surgical field.　For the second dose, patients were divided into two groups: Group A (antegrade cardioplegia, *n* = 10) and Group R (retrograde cardioplegia, *n* = 12).

In Group A, procedures included aortic valve replacement (AVR) in 3 patients (all combined with coronary artery bypass grafting [CABG]), mitral valve plasty (MVP) in 5, and mitral valve replacement (MVR) in 2 (including 1 with the maze procedure). In Group R, procedures included 7 cases of AVR (2 with CABG, 1 with MVR, and 1 with the maze procedure), 1 case of MVP with the maze procedure, and 4 cases of MVR (all with the maze procedure, including 1 with CABG). There were no significant differences between the two groups in age, height, weight, body surface area, or severity of aortic regurgitation (Table 1). Because the maze procedure increases postoperative creatine kinase (CK) levels due to ablation, these patients were excluded from CK analysis. Although both antegrade and retrograde cardioplegia were administered for approximately 3 min, minor differences in manual control of infusion initiation and termination occasionally resulted in small variations in total volume, particularly in the retrograde group where initial pressure equilibration required several seconds.

This study was conducted in accordance with the Declaration of Helsinki and approved by the Institutional Review Board of Fukuoka University (Approval No. U24-11-005). Written informed consent was obtained from all participants prior to data collection and analysis.

Thermography is a technique that visualizes infrared radiation detected by a thermal camera, with areas of higher radiation appearing red and lower radiation appearing blue. The captured infrared data are mathematically converted into temperature readings. The accuracy of thermographic measurements is reported within ± 2.0 °C or approximately 2% compared to a black body (emissivity = 1.0). Given that human skin has an emissivity of approximately 0.99, thermographic temperature readings are considered highly accurate [6]. However, interpretation must be cautious due to potential interference from background radiation and surface reflections. In this study, if a discrepancy was noted between the cardioplegia delivery temperature and the thermographic reading, recalibration was performed.

Thermographic measurements of the myocardial surface were obtained using a FLIR ONE Pro device (FLIR Systems, Inc., Wilsonville, OR, USA) positioned approximately 80 cm above the surgical field from the cranial side. If a significant discrepancy was observed between the cardioplegia delivery temperature and the thermographic reading, recalibration was performed within the measurement range. Measurements were taken on the anterior surface of the right ventricle, avoiding areas covered with adipose tissue, and the lowest surface temperature was recorded.

We evaluated the volume of cardioplegia administered, changes in myocardial surface temperature from the start to the end of delivery, and postoperative outcomes.

Our institution uses a blood cardioplegia system (MPS^®^2, Quest Medical, Inc., Allen, TX, USA) with the microplegia technique. The system includes two 50 mL cassettes: the ARREST AGENT CASSETTE (ARR) and the ADDITIVE CASSETTE (ADD). The ARR contains 2 mol/L potassium chloride (80 mEq/40 mL), while the ADD includes magnesium sulfate (40 mEq/40 mL), 2% lidocaine (100 mg/5 mL), nicorandil (5 mg/5 mL), and 10 units of rapid-acting insulin. Before arrest, cardioplegia was prepared by mixing ARR (22 mEq/L) and ADD (18 mL/L) with oxygenated blood (target potassium concentration: 26 mEq/L). After arrest, a maintenance dose was administered using ARR (12 mEq/L) and ADD (9 mL/L) over 5 min (target potassium: 16 mEq/L). The solution is not prepared ad hoc for each case but follows a reproducible protocol verified by our pharmacy and perfusion team.

For antegrade cardioplegia, the delivery temperature was maintained at 20 °C, with circuit pressure below 300 mmHg and a flow rate of 250–350 mL/min. The first and second doses were administered over 5 and 3 min, respectively, under time-based control. For retrograde cardioplegia, delivery temperature was also maintained at 20 °C, with tip pressure below 30 mmHg and a flow rate of 150–200 mL/min. The second dose was likewise administered over 3 min. Cardioplegia dosing was determined by infusion time (5 min for the initial dose, 3 min for the maintenance dose) under fixed flow and pressure settings, rather than by a fixed total volume.

**Table 1:** Patient characteristics and surgical procedures

	Group A (Antegrade, *n* = 10)	Group R (Retrograde, *n* = 12)	*P* value
Age (years)	70 (59–76)	73 (69.5–81.2)	n.s.
Gender male (%)	3 (30%)	6 (50%)	
HT (cm)	158 (148.2–167.1.2.1)	164.7 (156.9–170)	n.s.
BW (kg)	55.5 (48.6–65.4)	62.2 (54.3–73.6)	n.s.
BSA	1.56 (1.4–1.6)	1.71 (1.5–1.8)	n.s.
Grade of AR	0 (0–1.1.1)	0 (0- 0.75)	n.s.
Surgical procedure			
AVR	3 (CABG: 3)	7 (CABG: 2, MVR: 1, Maze: 1)	
MVP	5	1 (Maze: 1)	
MVR	2 (Maze: 1)	4 (Maze: 4, CABG: 1)	

HT = height, BW = body weight, BSA = body surface area, AR = aortic regurgitation

AVR = aortic valve replacement, MVP = mitral valve plasty, MVR = mitral valve replacement

CABG = coronary artery bypass grafting, Maze = maze procedure

Values are presented as median (interquartile range) unless otherwise indicated

Statistical analysis: Continuous variables are expressed as median (interquartile range). Intergroup comparisons were performed using Student’s t test or the Mann–Whitney U test, as appropriate. A p-value < 0.05 was considered statistically significant. All analyses were conducted using JMP version 14.0 (SAS Institute Inc., Cary, NC, USA).

## Results

**Fig. 1:**
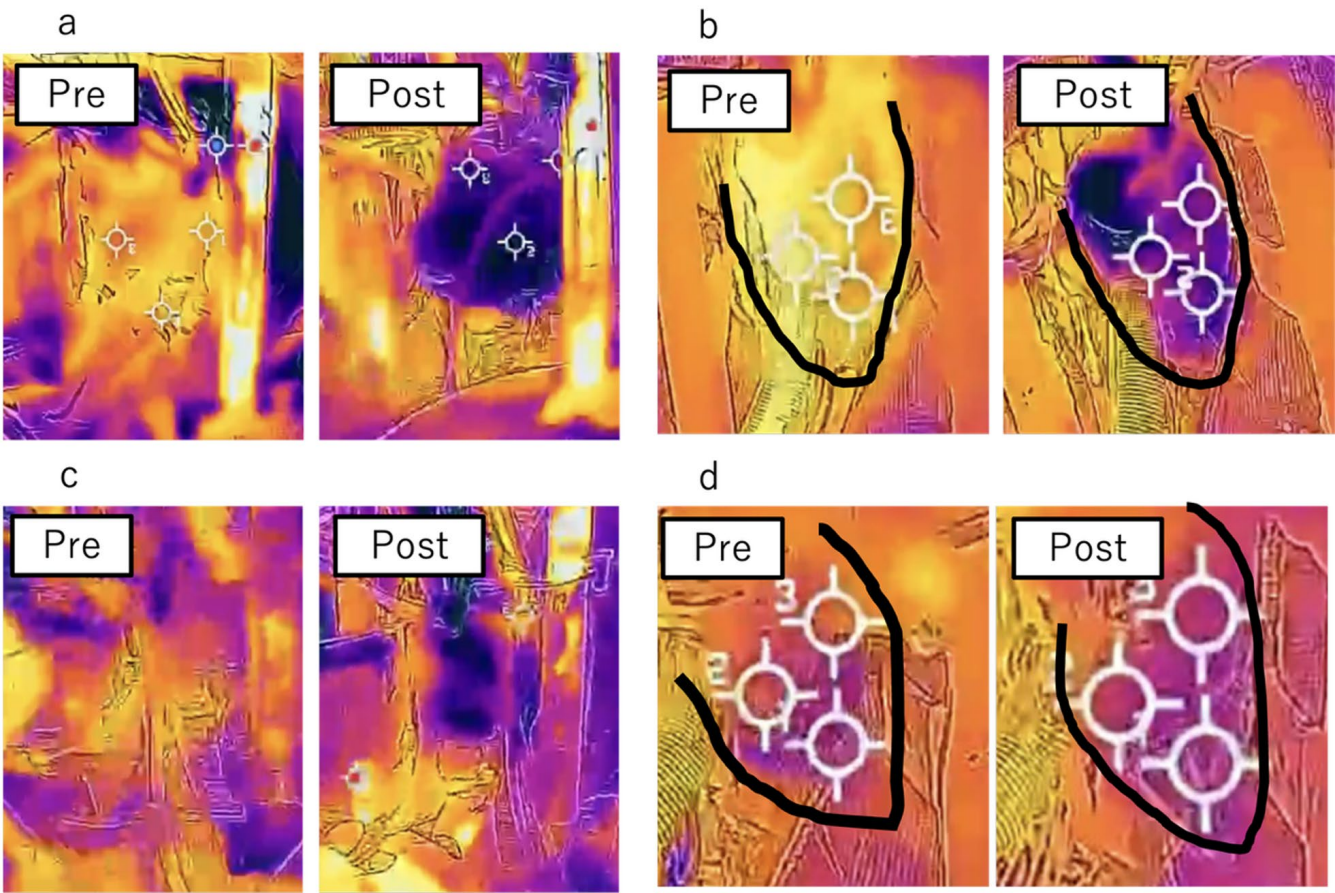
Representative thermography images

At the start of the first cardioplegia, the mean myocardial surface temperature was 31.6 ± 0.7 °C in Group A and 31.8 ± 1.4 °C in Group R. The mean cardioplegia volume was 2050 ± 396 mL in Group A and 2072 ± 251 mL in Group R, with no significant difference. With the delivery temperature maintained at 20 °C, myocardial surface temperature decreased to 22.7 ± 0.8 °C in Group A and 23.8 ± 1.6 °C in Group R after the first dose. As both groups received antegrade cardioplegia initially, no significant difference was observed (Fig. 1a and b).

At the start of the second cardioplegia, myocardial surface temperature was 27.6 ± 1.2 °C in Group A and 28.8 ± 1.7 °C in Group R. The mean cardioplegia volume was 1267 ± 333 mL in Group A and 1343 ± 642 mL in Group R, again with no significant difference. However, at the end of the second cardioplegia, myocardial surface temperature was significantly lower in Group A (22.7 ± 0.6 °C) than in Group R (24.6 ± 1.9 °C; *p* < 0.05) (Fig. 1c and d) (Table 2).

Figure 1a and b show thermography images before and after the initial administration of myocardial protection solution. Figure 1c shows images before and after the second administration of the anterograde cardioplegia, and Fig. 1d shows thermography images before and after the second administration of the retrograde cardioplegia.With in each figure panel, Pre indicates before administration of the cardioplegia, and Post indicates after administration.

**Table 2:** Myocardial surface temperature and cardioplegia volume

	Group A (Antegrade, *n* = 10)	Group R (Retrograde, *n* = 12)	*P* value
Cardioplegia volume at 1 st dose (mL)	2025 (1912–2100)	2100 (2000–2237)	n.s.
Myocardial temperature at 1 st cardioplegia start (℃)	31.6 (31.1–32.0)	31.5 (30.6–32.5)	n.s.
Myocardial temperature at 1 st cardioplegia end (℃)	22.5 (21.9–23.5)	23.3 (22.7–25.2)	n.s.
Cardioplegia volume at 2nd dose (mL)	1150 (1000–1525)	1175 (1012–1450)	n.s.
Myocardial temperature at 2nd cardioplegia start (℃)	28.1 (26.7–29.5)	28.2 (27.4–29.9)	n.s.
Myocardial temperature at 2nd cardioplegia end (℃)	22.8 (22.3–23.1)	23.9 (22.9–26.3)	< 0.05

Comparison of myocardial surface temperature and cardioplegia volume between Group A (antegrade cardioplegia) and Group R (retrograde cardioplegia) at each cardioplegia administration time point. Values are presented as median (interquartile range). n.s. = not significant.

Postoperative outcomes, including ejection fraction (EF), CK levels, catecholamine use, extubation time, hospital stay, and overall clinical outcomes, did not differ significantly between groups. There was no significant difference between the two groups in cardiopulmonary bypass time, aortic cross-clamp time, or reperfusion time; however, reperfusion time tended to be slightly longer in the R group. All patients were discharged in an ambulatory state (Table 3). Furthermore, we performed a subgroup analysis limited to valve procedures (*n* = 16). The trend of higher myocardial temperature in the retrograde group persisted, but statistical significance was not maintained due to the smaller sample.

**Table 3:** Intraoperative variables and early outcomes

	Group A (Antegrade, *n* = 10)	Group R (Retrograde, *n* = 12)	*P* value
Operative time (min)	295 (265.2–357.7.2.7)	293 (247–323)	n.s.
CPB time (min)	165 (138–185)	145 (133.7–184)	n.s.
ACC time (min)	117 (112.5–140.7.5.7)	104 (92.2–117)	n.s.
Reperfusion time (min)	23.5 (21.5–28.2)	26 (24.2–32.5)	n.s.
Peak CK level (U/L)	808 (718.5–966.5.5.5)	808 (465–945)	n.s.
Time to extubation (hours)	6.5 (5.1–10.2)	6.8 (5.5–11.3)	n.s.
Duration of catecholamine use (days)	1.3 (0.5–2.1)	1.4 (0.5–2.5)	n.s.
Pre-op EF (%)	71 (66–72.2.2)	66.5 (62.2–70.7)	n.s.
Post-op EF (%)	63 (60.2–66)	62 (60.5–63.7)	n.s.
Postoperative hospital stay (days)	13 (12–22.7.7)	14 (12.2–17.5)	n.s.

CPB = Cardiopulmonary bypass time, ACC = Aortic cross-clamp time

Values are presented as median (interquartile range). n.s. = not significant

## Discussion

Effective myocardial protection requires not only sufficient cardioplegia volume but also uniform distribution. Antegrade cardioplegia is the physiological and standard method; however, maldistribution may occur in patients with significant coronary stenosis. In hypertrophied myocardium, elevated vascular resistance may necessitate increased infusion pressure and volume. To reduce the risk of inadequate protection, our institution delivers cardioplegia based on infusion time rather than volume. We previously reported that 360 s of cardioplegia are required to cool the myocardium uniformly below 25 °C [5]. This finding suggests that administration time, not just volume, is critical to effective myocardial cooling. Thermographic monitoring of myocardial temperature may help confirm uniform cooling and enhance myocardial protection.

As shown in Table 2, the mean myocardial surface temperatures at the start of cardioplegia were 31.6 (31.1–32.0) °C in Group A and 31.5 (30.6–32.5) °C in Group R, both lower than the typical human core temperature of approximately 36.0 °C. A prior study using thermography to measure skin surface temperature [7] reported values ranging from 30.4 to 35.4 °C at an ambient room temperature of 34.0 °C. When the room temperature was lowered to 25.0 °C, skin temperature dropped to 27.2–34.1 °C, with lower readings in distal regions. At ambient temperatures below 25.0 °C, the chest surface temperature was approximately 32.6 °C. The myocardial surface temperatures observed at cardioplegia onset were comparable to these ranges, suggesting a likely influence from operating room ambient temperature.

Additionally, prior studies [8, 9] have shown that surface temperature in ischemic myocardial regions may decrease due to impaired blood flow during infarction, implying that some patients in this cohort may have had coronary artery stenosis or occlusion. In this study, the myocardial temperature at the end of the second cardioplegia was significantly lower in Group A than in Group R, indicating less effective myocardial cooling with retrograde delivery. Although previous studies identified 25 °C as a critical threshold for myocardial recovery in experimental settings, our data do not demonstrate any direct association between temperature levels and clinical outcomes. Therefore, this reference should be regarded as a physiological context rather than an outcome-based conclusion [10, 11].

Temperatures observed in Group R approached this threshold, raising concern about the adequacy of protection. The rationale for retrograde cardioplegia in this cohort was to maintain continuous surgical access during intracardiac procedures. No significant intraoperative or postoperative complications were observed in Group R. However, for procedures with extended cross-clamp times, the observed temperature gap may translate into clinically meaningful myocardial injury or impaired recovery.

This study has some limitations. It was conducted at a single institution with a limited number of cases, which may restrict the generalizability of the results. Because of the small sample size, some between-group differences (e.g., gender distribution, body weight) may not have reached statistical significance, and the results should be interpreted with caution. Therefore, this study was a pilot, exploratory analysis designed to evaluate the feasibility of thermographic assessment rather than to achieve definitive statistical power. The cardioplegia protocol, specifically the temperature, composition, and delivery method, reflects our institutional practice and may not represent techniques used at other centers. Additionally, although surface thermography is noninvasive and practical, it may not fully capture core myocardial temperature, especially in patients with hypertrophied or ischemic myocardium. Furthermore, differences in myocardial exposure and cooling conditions between valve replacement and CABG procedures may also affect thermography measurements. Larger, multicenter studies incorporating varied cardioplegia strategies are needed to validate these findings and further clarify their clinical relevance.

## Conclusion

This study demonstrates the practical application of intraoperative thermography for real-time assessment of myocardial surface cooling. Although retrograde delivery showed slightly less cooling than antegrade delivery, the study does not establish clinical superiority. Larger prospective studies are warranted to validate the physiological and clinical implications of these observations.

## Data Availability

No datasets were generated or analyzed during the current study.

## Abbreviations

AVR: Aortic Valve Replacement
CABG: Coronary Artery Bypass Grafting
CK: Creatine Kinase
EF: Ejection Fraction
LLM: Large Language Model
MPS: Myocardial Protection System
MVP: Mitral Valve Plasty
MVR: Mitral Valve Replacement
OR: Operating Room
